# Prevalence of HIV testing and associated factors among young adolescents in Eswatini: a secondary data analysis

**DOI:** 10.1186/s12887-022-03698-0

**Published:** 2022-11-14

**Authors:** Mi Sook Jung, Nondumiso Satiso Dlamini, Xirong Cui, Kyeongin Cha

**Affiliations:** College of Nursing, Chungnam National University, 266 Munhwa-ro, Jung-gu, Daejeon, 35015 South Korea

**Keywords:** Adolescent, HIV testing, Perception, Prevalence, Eswatini

## Abstract

**Background:**

HIV testing is a crucial starting point for prevention, early diagnosis, and treatment of HIV. Sub-Saharan Africa has the highest global HIV/AIDS prevalence and mortality, yet HIV testing remains sub-optimal. Thus, this study aimed to identify the prevalence of HIV testing and associated factors among young adolescents aged 10 to 14 years in Eswatini, a country with the highest HIV prevalence in the world.

**Methods:**

Data were obtained from Swaziland HIV Incidence Measurement Survey between 2016 and 2017 (SHIMS 2), an internationally supported national survey aimed at combating HIV/AIDS. A total of 739 young adolescents aged 10 to 14 years were selected for the final analysis after deleting cases with missing values for the key variables. The effects of demographic characteristics, HIV knowledge, HIV risk perception, belief about HIV testing, perceived service accessibility, and parent-child sexual and reproductive health communication on lifetime HIV testing as an outcome variable, were explored using multivariable logistic regression.

**Results:**

Only 52.0% of young adolescents reported “ever tested” for HIV in their lifetime. Age (OR = 0.81, 95% CI = 0.73–0.90), residence (OR = 0.56, 95% CI = 0.43–0.74), and perceived service accessibility (OR = 3.10, (95% CI = 1.47–6.56) were identified as important factors associated with receiving HIV testing among young adolescents.

**Conclusions:**

A low rate of HIV testing was identified among young adolescents in Eswatini compared to the intended global goal of HIV testing coverage. Our findings suggested the importance of young adolescent-friendly educational and environmental interventions needed to improve the prevalence of HIV testing by reducing misperceptions about the risk of HIV and alleviating environmental constraints to access to HIV services.

**Supplementary Information:**

The online version contains supplementary material available at 10.1186/s12887-022-03698-0.

## Background

HIV testing is a crucial starting point for prevention, early diagnosis, and treatment of HIV to increase survival rate, improve quality of life, and preclude further transmission [1, 2]. In order to improve motivation toward maximizing HIV testing coverage, which is an optimal measure to achieve an HIV-free generation, the Joint United Nations Programme on HIV/AIDS (UNAIDS) outlined a global target named 90–90-90 in 2014. The 90–90-90 target aimed to ensure that 90% of the people living with HIV (PLHIV) should be diagnosed and know their HIV status, 90% of people living with HIV should be initiated on antiretroviral therapy (ART), and 90% of those started on ART should have their viral load suppressed by 2020 [3]. The 90–90-90 target was further accelerated to 95–95-95 by 2030, following the remarkable progress made by the UNAIDS and other global stakeholders in achieving the 90–90-90 targets in the general population of individuals aged 15–49 years in a couple of countries globally including the Sub-Saharan Africa by 2020 [4–6]. In 2020, Eswatini was the first country in Africa to achieve the 95–95-95 target [6, 7]. However, considering the disparities in accessing HIV prevention and treatment interventions [8], it is not clear whether this success can be attributed to young adolescents.

Sub-Saharan Africa is a region with the highest prevalence rate of HIV among adolescents and young adults [9]. Considering the high prevalence of HIV and the anticipated increased risk of transmission in a high HIV-burdened country like Eswatini, the HIV management guidelines of Eswatini suggested that young adolescents should be tested for HIV at least once a year depending on risk assessment [10]. However, the latest report by United Nations Children’s Fund (UNICEF) showed that approximately 88% of all HIV-infected adolescents aged 10 to 19 years live in Sub-Saharan Africa, yet only 17 to 25% of this age group had tested for HIV in the past year in 2020 [11]. Similarly, another review study with data from 18 countries in Sub-Saharan Africa also reported the low rate of HIV testing among adolescents aged 15 to 19 years, showing 2–24% in male adolescents and 4–44% in female adolescents across countries [12]. According to a recent analysis of data from PHIA surveys conducted in seven countries, including Eswatini, about 39% of children and young adolescents (1–14 years) living with HIV were undiagnosed, meaning they were unaware of their HIV status [13]. Low rates of HIV testing can lead to a reduction of early treatment, resulting in insufficient support and increasing mortality in adolescents living with HIV [8, 14].

Considering that HIV-related mortality has ranked as a top killer in Sub-Sahara African adolescents between the ages of 10 and 19 years [15], these young adolescents should be prioritized early in the development life cycle before being exposed to environmental and societal factors with potentially damaging consequences for sexual and reproductive health [16]. However, sufficient attention has not been paid to young adolescents. This gap is probably related to limited age-specific data to identify potential factors for facilitating the availability, accessibility, and utilization of HIV testing services [17]. A systematic review on HIV testing among children and adolescents in the Sub-Saharan region highlighted that data from existing literature had not been adequately stratified by age [17]. For this reason, strategies used in HIV testing and treatment that were developed for older adolescents and young adults were mostly repeated with little consideration for the needs of young adolescents [17–20]. As a result, HIV-related mortality varied with age [14]. Only a 10% reduction was reported among adolescents aged 10–19 years while 74 and 64% reductions were found in children aged 0–9 years and other age groups, respectively [21].

According to existing literature, HIV testing uptake among adolescents and young people was associated with several factors, including age, gender, residence, HIV knowledge, and risk perception, stigma, HIV-related belief, accessibility to HIV testing services, and prior experience to participate in sexual reproductive health (SRH) communication [18, 22–27]. Despite the valuable information from previous studies regarding the low rate of HIV testing and associated factors, further investigations are needed to determine the relevance of what is given from different age groups as factors that can play the same role in explaining the HIV testing uptake in young adolescents. Young adolescence was highlighted as a critical target population for the prevention and treatment of HIV to achieve the global target of combating HIV by 2030 [28]. Therefore, this study aimed to identify the prevalence of HIV testing uptake and examine the factors associated with HIV testing among young adolescents aged 10 to 14 years in Eswatini.

## Methods

### Research design and sample

This study is a secondary data analysis obtained from the Swaziland HIV Incidence Measurement Survey (SHIMS 2). This nationally representative survey was conducted between September 2016 and March 2017 to gather demographic and HIV-related data as part of the multicounty Population-based HIV Impact Assessment (PHIA) project. To obtain participants for SHIMS 2, a two-stage, stratified cluster sample design was used. The sample frame for this survey included all households in Eswatini, estimated at 212,195 homes, including 2064 enumeration areas (E.A.) based on the country’s 2007 census of population and housing. In the first sampling stage, the sample was stratified by urban or rural status within four regions (Hhohho, Manzini, Lubombo, and Shiselweni) of Eswatini, and a total of 287 EAs were selected with probability proportionate to the number of households. The second stage involved a random selection of homes from each stratum using the equal probability method, which rendered an average of 20 households selected from each cluster. Children were eligible to participate if they resided in a selected household and slept in the house the night before the interview. Households were eligible for selection if 1) they were known households during the time of listing, 2) occupied at the time of the interview, and 3) vacant dwelling units that could potentially be occupied at the time of the interview. Before data collection, consent from parents or guardians of children 0 to 14 years was sought to approach a minor, and assent was sought from children 10 to 14 years whose parents or guardians had consented to their participation.

**Fig. 1:**
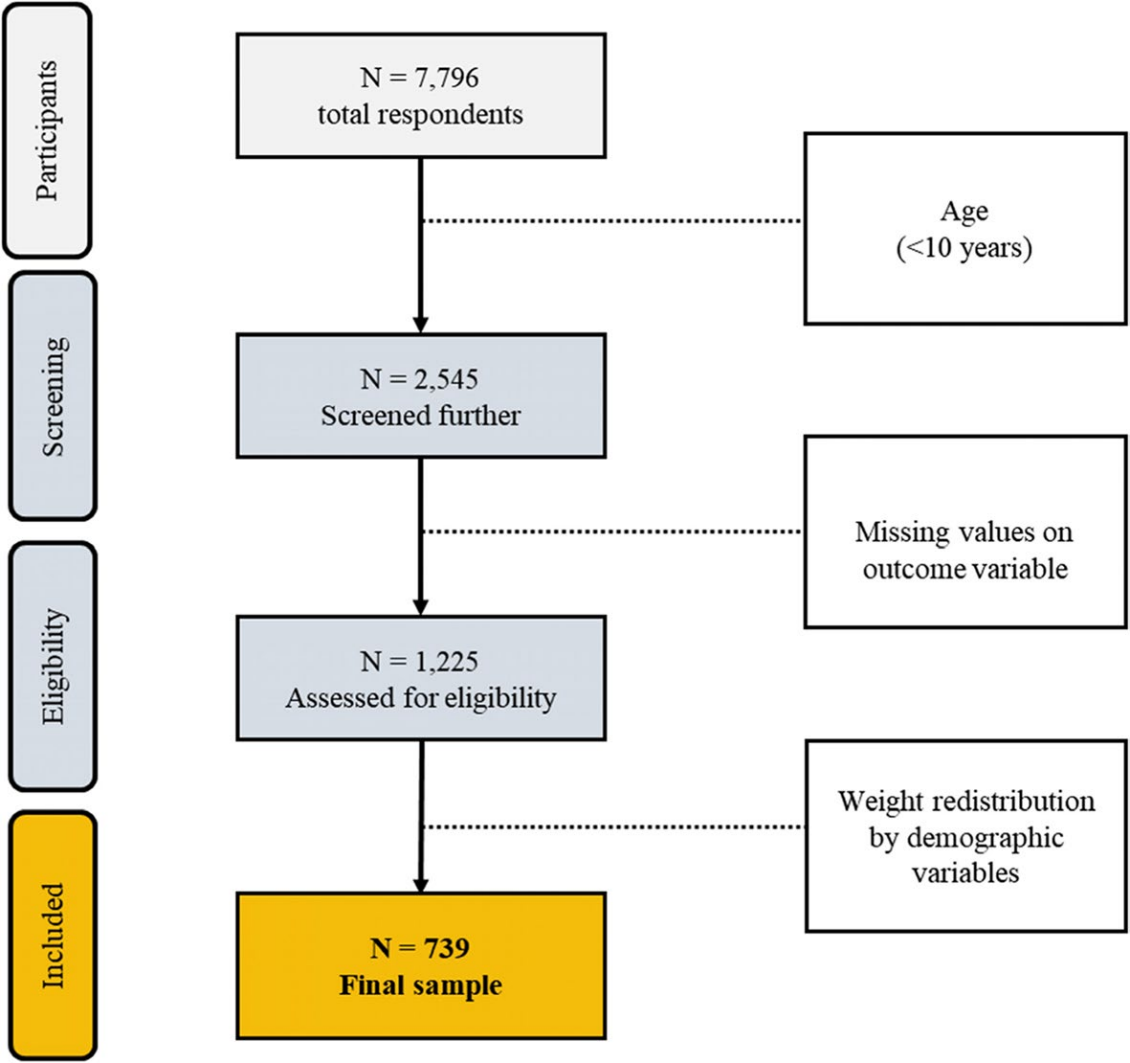
A flow of sample selection

The original data set contained 7796 children aged 0 to 14 years [29]. For the purposes of this study, we selected data from those who were adolescents aged 10 to 14 years and had provided information about HIV testing and key variables including age, gender, residence, educational level, HIV knowledge, HIV risk perception, HIV test-related belief, HIV testing service accessibility, and parent-child SRH communication. After deleting respondents younger than 10 years, a sample of 2, 545 respondents remained. The additional exclusion was made when individuals had missing values on the outcome variable and other independent variables selected for purpose of this study. Finally, a sample size of 739 was included in the analysis, and weights were attached to account for the respondents with missing or incomplete values for the demographic variables of each respondent. A sample flow chart is presented in Fig. 1. below.

### Measures

HIV testing was selected as an outcome variable in this study. Independent variables included HIV knowledge, HIV risk perception, HIV test-related belief, HIV testing service accessibility, and parent-child SRH communication. Sociodemographic factors including age, gender, residence, and educational level were evaluated to define the sample characteristics.

HIV testing was assessed using one question on individuals’ past experience of getting an HIV test: “Have you ever tested for HIV?” This question was answered with “yes” or “no.”

HIV knowledge was measured as a categorical variable by asking 8 questions to define comprehensive knowledge about HIV. Participants were asked to respond “yes” or “no” to each question. The score of HIV knowledge ranged from 0 to 8, with higher scores interpreted as more accurate knowledge [29, 30]. If participants had 0 or 1 correct answer to the questions, they were classified into the group with a low level of HIV knowledge. If participants had 2 to 5 correct answers, they were considered the group with middle-level HIV knowledge. If participants answered 6 or more questions correctly, they were categorized as a high knowledge level group.

HIV risk perception was measured by using one question to assess the perceived risk of HIV infection: “How likely do you think it is for you to get HIV?”. The participants answered the question by selecting one of four options, including “likely,” “somewhat likely,” “unlikely,” and “already know my HIV status”. Individuals who answered with “already know my status” were excluded from the analysis of HIV risk perception because, according to the SHIMS 2 codebook, these were individuals who had already tested positive for HIV. To simplify the presentation and interpretation of the results, this variable was converted into a categorical variable by splitting the scale using the median as a cut-off point to re-define the high and low groups in this study [30–32]. Participants with 1 or less scores were classified into the group with lower HIV risk perception, and those who scored 3 and above were classified into the high HIV risk perception group.

HIV test-related belief was measured as a categorical variable by asking one question on individuals’ beliefs about HIV testing: “Should everyone get tested for HIV?” This question was answered with “yes” or “no” [29].

Parent-child SRH communication was measured as a categorical variable by asking participants 3 questions to define young adolescents’ openness to discuss sex-related topics with their parents or guardians. These questions included, “Have you ever discussed HIV with your parent or guardian?”, “Have you ever talked to parent/guardian about sex?” and “If you have a problem, can you freely discuss it with your parent or guardian?” They were asked to respond “yes” or “no” to these questions. The selection of these three items and classifying them as “parent-child SRH communication” single variable was based on previous studies which demonstrated a close relationship between these items [33, 34]. A sum score including all three items was computed. If participants had a score of less than 2, then that group was classified as “low”, meaning they could not freely talk about SRH related issues with parents or caregivers. If participants had a score of 2 and above, they were classified as the “high”, meaning they could freely talk about sexual and reproductive health issues with their parents/caregivers.

Perceived service accessibility was measured as a categorical variable to assess individuals’ perceived ease or difficulty to access HIV testing services: “If you wanted to, could you get an HIV test”? Participants were asked to respond “yes” or “no” [29]. An additional questionnaire file shows this in more detail (see Additional file 1).

### Statistical analyses

All data analyses were conducted in SPSS version 26. Descriptive statistics, including frequency, mean and standard deviations, were used to describe the sample characteristics. Chi-square tests were conducted to examine the differences in HIV testing by HIV knowledge, HIV risk perception, HIV test-related belief, parent-child SRH communication, and perceived service accessibility and sociodemographic factors including age, gender, and residence. Multivariable logistic regression model was developed to determine the factors associated with HIV testing. In this model, eight independent variables were selected based on theoretical and statistical considerations [22–25] as possible predictors of HIV testing; a) age as a continuous variable; b) gender, c) residence, d) HIV-test related belief, e) perceived service accessibility, and f) HIV risk perception as categorical variables; g) HIV knowledge and h) parent-child SRH communication as continuous variables. All analyses in this study were performed using sample weights and complex sample approaches to account for the survey design used by SHIMS 2 [35].

## Results

### Sociodemographic characteristics

Sociodemographic characteristics of 739 respondents are presented in Table 1. All participants were young adolescents whose ages ranged from 10 to 14 years, with an average age of 12.14 years. More than half of them were females, resided in urban areas, and were in primary school at the time of data collection. Interestingly, those who reported having ever tested for HIV were only 52.00%, with a higher HIV testing rate observed in females than males.

**Table 1:** Weighted sample distribution by sociodemographic characteristics (*N* = 739)

Variables	*n* (%)	M (SE)
Age (Years)		12.14 (0.04)
Gender		
Male	342 (46.30)	
Female	397 (53.70)	
Residence		
Rural	277 (37.50)	
Urban	462 (62.50)	
Level of education		
Primary School	618 (83.60)	
High School	116 (15.80)	
HIV testing		
Ever tested	384 (52.00)	
Never tested	355 (48.20)	

### Differences between HIV testing and independent variables

As presented in Table 2, HIV testing in a lifetime differed significantly depending on the residence, level of education, service accessibility, and parent-child SRH communication. Individuals residing in rural areas had fewer chances of getting an HIV test than those in urban areas (*χ* ^2^_=_ 11.98_,_ *p* < .001). Individuals in the lower grades were less likely to have ever tested for HIV than those in higher grades (*χ* ^2^ = 10.84, *p* < .001). Individuals with perceived ease of accessibility to testing services were more likely to have ever tested for HIV test than those with difficulty accessing HIV testing services (*χ* ^2^ = 14.80, *p* < .001). Young adolescents with experience of having parent-child SRH communication with their parents or guardian were more likely to report having ever tested for HIV than those who did not (*χ* ^2^ = 5.72, *p* = .02). HIV testing did not significantly differ by gender, HIV knowledge, and HIV test-related belief in this analysis.

**Table 2:** Weighted differences between HIV testing and independent variables among participants

Variables	HIV testing		*χ*^2^
	Yes	No	
Gender			
Male	186 (48.3)	157 (44.1)	1.22
Female	199 (51.7)	198 (55.9)	
Residence			
Rural	168 (43.6)	109 (30.8)	11.98^***^
Urban	217 (56.4)	246 (69.2)	
Level of education			
Primary School	333 (86.6)	285 (80.3)	10.84**
High School	47 (12.2)	70 (19.7)	
HIV Knowledge			
Low	17 (4.4)	23 (6.6)	2.99
Middle	132 (34.5)	134 (37.9)	
High	234 (61.1)	197 (55.5)	
HIV Risk Perception			
Low	336 (87.4)	300 (84.5)	1.26
High	48 (12.6)	55 (15.5)	
HIV test-related belief			
No	12 (3.1)	13 (3.7)	.20
Yes	372 (96.9)	342 (96.3)	
Service accessibility			
No	12 (3.1)	36 (10.3)	14.80^***^
Yes	372 (96.9)	318 (89.7)	
Parent-child SRH communication			
No	238 (62.1)	251 (70.7)	5.72^*^
Yes	146 (37.9)	104 (29.3)	

*Abbreviation*: *SRH* sexual and reproductive health

*Note*: ^*^p < .05, ^**^p < .001

### Multivariable logistic regression analysis

The results of multivariable logistic regression analysis are presented in Table 3. Getting tested for HIV was significantly associated with age, residence, and perceived service accessibility. One unit increase in age was associated with 0.81 times (95% CI = 0.73–0.90) higher odds of getting an HIV test. Individuals living in urban areas had 5.6% higher odds of getting tested (OR = 0.56, 95% CI = 0.43–0.74) than those in rural areas. The OR for adolescents who could access HIV testing was 3.10 (95% CI = 1.47–6.56) compared to those who could not. Parent-child SRH communication was not significantly associated with HIV testing uptake. Similarly, HIV knowledge, HIV risk perception, and HIV test-related belief were not significant factors of HIV testing for young adolescents.

**Table 3:** Multivariable logistic regression model

Variables	B (SE)	OR (95% CI)
Age	0.21 (.05)	0.81 (0.73–0.90)^**^
Gender (female)	0.24 (.16)	0.79 (0.57–1.09)
Residence (urban)	0.58 (.14)	0.56 (0.43–0.74)^**^
Sum of HIV knowledge	0.87 (.89)	2.38 (0.40–14.00)
HIV Risk perception (high)	0.19 (.29)	1.21 (0.68–2.15)
HIV test-related belief (yes)	0.03 (.43)	1.03 (0.43–2.44)
Perceived service accessibility (yes)	1.13(.38)	3.10 (1.47–6.56)^*^
Parent-child SRH communication	0.30 (.31)	1.47 (0.80–2.70)

*Abbreviations*: *SRH* sexual and reproductive health, *OR* Odds Ratio, *CI* Confidence Interval

*Note*: ^*^ *p* < .05, ^**^ *p* < .001

## Discussion

Eswatini made remarkable progress in HIV testing coverage among the general population of individuals aged 15 to 49 years and was applauded for being among the first countries to reach the global target of the first 90% along with other countries like Botswana and Namibia in Africa in 2018 [5]. In 2020, Eswatini was the first country in Africa to reach the 95–95-95 global target [7]. However, it was difficult to say that the same progress was obtained in the age group of young adolescents. This study found that the lifetime HIV testing rate among young adolescents aged 10 to 14 years in Eswatini was 52% compared to 98% in the general population [7], indicating a huge gap between the rate in this study and the goal proposed by the global targets. This age-related hidden gap may be of serious concern and highlights the need for greater participation of marginalized young adolescents who may be missed out on HIV response programs as some may be unknowingly infected hence delaying enrolment into care [16].

HIV testing uptake was significantly associated with greater access to HIV testing services in this study. As anticipated perceived accessibility to HIV testing services was significantly associated with HIV testing uptake among young adolescents. Respondents who reported difficult accessibility to HIV testing services were less likely to have received an HIV test in a lifetime. This finding is consistent with previous studies showing that service accessibility issues were associated with lower participation in HIV testing among adolescents [24, 28]. To bridge the gap in receiving HIV tests by dealing with barriers to accessibility among young adolescents, introducing adolescent-friendly health services (AFHS) within the communities in which young adolescents reside was recommended [36, 37]. The AFHS was defined as an evidence-based and specialized approach to addressing health system barriers and catering to developmental needs by providing a more accessible, acceptable, equitable, appropriate, and effective social environment for young people [38]. A recently published systematic review study supported that AFHS in health facilities and clinical settings positively influenced health service utilization for HIV testing and other sexually transmitted diseases or performing preventive behaviors in Sub-Saharan Arica [39]. However, there is a lack of understanding and limited knowledge in the literature regarding implementing appropriate strategies for delivering AFHS to help young adolescents receive HIV testing in resource-constrained out-of-facility settings [39]. Considering the effectiveness of AFHS in meeting adolescents’ sexual and reproductive health needs, future research is needed to investigate how AFHS can be applied over an extended period in marginalized communities to achieve the desired goals, especially in ending HIV.

HIV risk perception is vital in determining health behavior practices such as routine HIV testing as prevention. However, HIV testing uptake was not significantly associated with higher risk perception for HIV infection in this study. More than 80% of young adolescents reported lower HIV risk perception regardless of HIV test status. Although young adolescents who reported lower HIV risk perception were more likely to get an HIV test than those with higher HIV risk perception, the difference did not reach the significance level. Interestingly, this age group tends to believe that they are at low risk of contracting HIV, and their misperception about HIV transmission may increase the likelihood of engaging in risky sexual behaviors and their vulnerability to HIV infection [36]. Similarly, a couple of other studies conducted among African adolescents showed that adolescents and young people were likely to have incorrect HIV risk perception such that even those at high risk of HIV acquisition had lower HIV risk perception [27, 36, 37]. Therefore, future research is required to understand better the drivers of HIV risk perception among young adolescents.

There is a growing literature showing that young adolescents who freely communicated about sex-related topics and HIV with their parents or caregivers were likely to practice safer sex behaviors, including HIV prevention [40]. Contrary to these findings, the association between parent-child SRH communication and HIV testing uptake was not significant in this study. This unexpected inconsistency in the effect of parent-child SRH communication on health behavior practices and HIV prevention may be associated with the characteristics of communications and the individuals involved in SRH communication. First, a meta-analysis study suggested that the frequency, content, or how pleasant the conversations were likely to influence the effectiveness of the parent-child SRH communication among adolescents [40, 41]. Our data did not provide detailed information about the nature of the communication according to the above-mentioned factors, except for the knowledge that some adolescents engaged in SRH communication with parents or caregivers. Second, significant differences in the effect of communication on healthy behaviors such as HIV prevention were found between the gender of the adolescents and parents or caregivers, with more substantial effects observed in girls than in boys. Parent-child SRH communication between mothers and daughters was more significantly associated with positive outcomes compared to father-son communication [40, 42, 43]. It is necessary to consider that the interaction between the nature of SRH communication and the gender of the individuals involved may have different effects on communication outcomes [33]. Therefore, robust and well-designed future research on the determinants of parent-child SRH communication’s positive effects on healthy behavior practices and HIV prevention among young adolescents is recommended.

### Strengths and limitations

The strength of this study is that it is the first-ever study to provide information about the prevalence of HIV testing and associated factors among young adolescents in Eswatini and probably in the low- and middle-income countries highly burdened by HIV. The findings of this study can inform policymakers to plan timely and effective interventions that will accelerate remarkable progress of HIV testing coverage among young adolescents. However, this study also had some limitations. First, this study is that it only focused on individual factors that influence HIV testing among young adolescents. However, to achieve maximum HIV testing coverage, there is a need to apply ecological approaches of integrating other factors, including environmental, community, organizational, and societal level factors, which were not addressed in this research. Hence, future research must consider examining the influence of such factors on HIV testing uptake among young adolescents. Second, this analysis did not present a comprehensive model for factors related to HIV testing uptake due to the lack of data that could be explained by sociocultural factors of Eswatini, a high HIV-burdened country, such as parent’s HIV status, information from parents/guardians regarding previous children’s HIV testing, and the lack of information about the barriers of HIV testing among young adolescents. Future research needs to consider the application of a tested and validated conceptual model when examining the factors associated with HIV testing among young adolescents and across different age groups.

## Conclusion

This study shows that HIV testing among young adolescents in Eswatini is significantly lower than the global targets. Our findings highlighted some important factors associated with HIV testing among young adolescents, including increasing age, urban residence, and higher awareness of the ease of access to testing services. International organizations, national policymakers, and healthcare professionals need to carefully consider these factors and other differentiated approaches that cater to differences in personal characteristics and external resources when developing appropriate strategies to reduce possible gaps in receiving HIV tests. Additionally, primary research on the barriers to HIV testing is recommended to expand understanding and planning of programs to alleviate barriers and further research to provide more data about the prevalence of HIV testing and gender-specific associated factors among young adolescents. Age-and-gender specific, practical, and culturally appropriate strategies should be considered when developing interventions to enhance the uptake of HIV testing among young adolescents.

## Supplementary Information

**Additional file 1.** Questionnaire.

## Data Availability

The datasets used and analyzed in the current study are available upon request from the corresponding author.

## Abbreviations

AFHS: Adolescent-Friendly Health Services
AIDS: Acquired Immunodeficiency Syndrome
ART: Anti-Retroviral Therapy
CI: Confidence interval
EA: Enumeration Areas
HIV: Human Immunodeficiency Virus
OR: Odds Ratio
PHIA: Population-based HIV Impact Assessment
PLHIV: People Living with HIV
SHIMS: Swaziland HIV Incidence Measurement Survey
SRH: Sexual Reproductive Health
UNAIDS: Joint United Nations Programme on HIV/AIDS
UNICEF: United Nations Children’s Fund
