# Supplementary material for: Prevalence of HIV testing and associated factors among young adolescents in Eswatini: a secondary data analysis
Source: BMC Pediatr. 2022 Nov 14;22:659. doi: 10.1186/s12887-022-03698-0 (PMC9661805; doi:10.1186/s12887-022-03698-0)
Supplement: Supplementary file 1 — Additional file 1. Questionnaire. [file 12887_2022_3698_MOESM1_ESM.docx]

Questionnaire

**Sociodemographic variables**

Age

Gender 1) Male 2) Female

Residence 1) Shiselweni 2) Lubombo 3) Manzini 4) Hhohho

Educational level 1) Primary school 2) Secondary school

**Independent variables**

***HIV knowledge***

| 1. | Can a person reduce their chance of getting HIV by not having sex?  1. yes 2. No |
| --- | --- |
| 2. | Can a person reduce their chance of getting HIV by using condoms when having sex?  1. yes 2. No |
| 3. | Can a healthy-looking person have HIV or AIDS?  1. yes 2. No |
| 4. | Can a mother with HIV or AIDS pass HIV to her unborn baby?  1. yes 2. No |
| 5. | Are there medicines that people with HIV or AIDS can take to help them live longer?  1. yes 2. No |
| 6. | Can ARVs make people with HIV less likely to spread the virus?  1. yes 2. No |
| 7. | Can a person get HIV from a mosquito bite?  1. yes 2. No |
| 8. | Can male circumcision help prevent HIV infection?  1. yes 2. No |
|  | ***HIV-related belief*** |
| 13. | Should everyone get tested for HIV?  1. yes 2. No |
|  | ***Parent-child SRH communication*** |
| 16. | Have you ever discussed about HIV with your parents or guardian?  1. yes 2. No |
| 17. | If you have a problem, can you freely go to your parent or guardian for help?  1. yes 2. No |
| 18. | Have you ever discussed about sex with your parents?  1. yes 2. No |
|  | ***Accessibility to services*** |
| 19. | If you wanted to, could you get an HIV test?  1. yes 2. No |
|  | ***HIV risk perception*** |
| 20. | How likely do you think it is for you to get HIV?  1.Likely 2. Somewhat 3. likely 4. Already positive |
|  | **Outcome variable*; HIV testing*** |
| 22. | Have you ever been tested for HIV?  1. yes 2. No |
